# Electronic Properties and Carrier Trapping in Bi and Mn Co-doped CsPbCl_3_ Perovskite

**DOI:** 10.1021/acs.jpclett.0c01567

**Published:** 2020-06-17

**Authors:** Damiano Ricciarelli, Edoardo Mosconi, Boualem Merabet, Olivia Bizzarri, Filippo De Angelis

**Affiliations:** † Department of Chemistry, Biology and Biotechnology, University of Perugia, Via Elce di Sotto 8, 06123 Perugia, Italy; ‡ Istituto CNR di Scienze e Tecnologie Chimiche “Giulio Natta” (CNR-SCITEC), Via Elce di Sotto 8, 06123 Perugia, Italy; § Faculty of Sciences and Technology, University of Mustapha Stambouli, Mascara 29000, Algeria; ∥ Laboratoire de Physique Computationnelle des Materiaux, Faculté de Sciences Exates, Deṕartement de Physique, Université Djillali Liabes̀, Sidi Bel Abbes̀ 22000, Algeria; ⊥ CompuNet, Istituto Italiano di Tecnologia, Via Morego 30, 16163 Genova, Italy

## Abstract

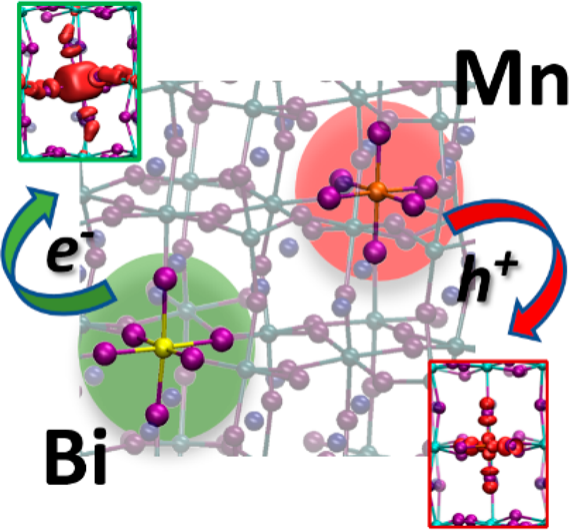

Metal halide perovskites exhibit impressive optoelectronic properties with applications in solar cells and light-emitting diodes. Co-doping the high-band gap CsPbCl_3_ perovskite with Bi and Mn enhances both material stability and luminescence, providing emission on a wide spectral range. To discuss the role of Bi^3+^ and Mn^2+^ dopants in tuning the CsPbCl_3_ perovskite energy levels and their involvement in carrier trapping, we report state-of-the-art hybrid density functional theory calculations, including spin–orbit coupling. We show that co-doping the perovskite with Bi and Mn delivers essentially the sum of the electronic properties of the single dopants, with no significant interaction or the preferential mutual location of them. Furthermore, we identify the structural features and energetics of transitions of electrons trapped at Bi and holes trapped at Mn dopant ions, respectively, and discuss their possible role in determining the optical properties of the co-doped perovskite.

Although metal halide perovskite semiconductors^1,2^ have recently emerged as inexpensive absorber layers in solar cells,^3−5^ these materials have also shown high mobility,^6−8^ narrow band emission, a tunable band gap,^9−12^ photon recycling,^13^ and bright emission,^14^ features that are appealing for solid state lighting applications. As an example, the CsPbCl_3_ perovskite has an appropriate band gap for exciton energy transfer^15^ and exhibits excellent optical properties like a narrow emission band, a wide color range, and overall promising optoelectronic applications.^16^ However, this material may suffer from the low photoluminescence quantum yield of the blue–violet radiation that it emits (still <10%).^17^ Doping lead halide perovskites with different metal ions is an effective approach to tuning their optical, electronic, and magnetic properties,^18^ through energy or charge transfer interaction between the host and dopant.^19^ Very recently, by partial substitution of Pb sites with Bi^3+^ in all-inorganic cesium lead bromide perovskites, Miao et al.^20^ found the resulting material to show an enhanced absorption over the entire visible spectrum together with a low trap density and a high carrier mobility. Hu et al.^21^ succeeded in stabilizing the α phase of CsPbI_3_ by incorporating Bi^3+^ ions into the perovskite host. The doped compound exhibited enhanced photoelectric performance and moisture stability compared to those of the pure perovskite. Snaith and co-workers pointed out by means of ^207^Pb NMR and ellipsometry spectroscopy the band gap of MAPbI_3_ perovskite to be not significantly affected by the introduction of the Bi^3+^ dopant, which solely contributes to the increase in the number of defects in the material.^22^ Some of us confirmed this result through density functional theory (DFT) computational analyses highlighting the presence of deep traps associated with Bi in doped MAPbI_3_ perovskites, which are responsible for the modified optical properties.^23^ Kang et al.^24^ found that the free electrons that originated from Bi^3+^ doping in CsPbCl_3_ are significantly compensated by the formation of native acceptor defects, because the Bi_Pb_ substitutional defect (bismuth replacing lead in the crystal lattice) predominantly exists in the 1+ charge state. In a recent investigation of Bi-doped MAPbBr_3_ thin-film perovskites, Ulatowski et al. observed an enhancement of electron trapping by defects with a resulting ultrafast charge carrier decay in the IR region (∼1.2 eV), possibly associated with the presence of bromine interstitials that originated from compensation of the Bi^3+^ charged dopant.^25^

Mn^2+^ doping has been widely investigated over the past three decades in the case of semiconductor quantum dots, such as ZnS, ZnSe, CdSe, and ZnO, with the purpose of obtaining luminescence in the orange region of the spectrum.^26−28^ The incorporation of Mn^2+^ into semiconducting nanocrystals provides the emergence of a broad photoluminescence peak at ∼600 nm that is widely attributed to a spin-forbidden transition arising from the decay of the ^4^T_1_ (t_2g_^4^e_g_^1^) excited state of Mn^2+^ to the ^6^A_1_ (t_2g_^3^e_g_^2^) ground state.^29−31^ Significant efforts were made to understand more about the excitation and de-excitation processes related to the Mn^2+^ dopant. It is generally accepted that the excited state of the Mn^2+^ dopant is activated by impact excitation from the optically excited carriers of the semiconducting host to the dopant ion, thus generating the ^4^T_1_ state by energy transfer.^32−35^ Auger recombination has also been demonstrated as a de-excitation pathway under electrochemically controlled charging conditions.^36^ Very recently Gahlot et al. performed time-resolved spectroscopy studies that support the involvement of a transient Mn^3+^ species that was proposed to mediate the excitation of Mn^2+^ in Cd_*x*_Zn_1–*x*_Se quantum dots.^37^

Mn^2+^ doping has been also successfully implemented in metal halide perovskites.^38^ Despite the difference between the ionic radius of Pb^2+^ (∼133 pm) and that of Mn^2+^ (∼57 pm), which is responsible for lattice contraction, the stability of doped perovskite nanocrystals was much the same as that of undoped ones.^15^ The typical Mn^2+^ emission is still observed in CsPbX_3_ perovskites with features similar to those of conventional Mn-doped quantum dots. Interestingly, it was found that the nature of the X halide impacts the quantum yield of the dopant photoluminescence, with CsPbCl_3_ delivering the maximum quantum efficiency.^15^ Pandey et al. investigated the band structure of Mn:CsPbCl_3_, finding Mn 3d orbitals within the perovskite band gap, which could contribute to the observed luminescence.^39^ A similar band structure was found by Pradeep et al. for Mn:CsPbBr_3_ perovskite, additionally revealing a significant phonon coupling associated with Mn and Pb modes related to dopant/host charge transfer.^40^ The typical photoluminescence due to Mn was detected also in CH_3_NH_3_Pb_*x*_Mn_1–*x*_Cl_3_ nanocrystals that show Mn^2+^ dopant emission at ∼610 nm with a high quantum yield.^18^ Mn-doped CsPbCl_3_ shows an optimal photoluminescence quantum yield at low Mn doping and exhibits an emission centered at ∼590 nm.^41^ In addition to tuning the optoelectronic properties, introducing Mn^2+^ ions into the perovskite was shown to significantly stabilize the crystal lattice, as illustrated by Akkerman et al. for CsPb_*x*_Mn_1–*x*_I_3_ nanocrystals.^42^

Overall, these results indicate^43^ that replacing Pb^2+^ with different metal ions such as Mn^2+^ (orange-red emission^44^) and Bi^3+^ (blue emission^45,46^) is successful for covering a wide luminescence range.^43^ Practically white emission can be obtained by a co-doping with such dopant metal ions.^47^ Shao et al.^9^ reported that dual ion Bi^3+^/Mn^2+^ co-doping of the CsPbCl_3_ perovskite facilitates stable multicolor and white light emission, exhibiting tunable emission spanning the wide range of correlated color temperature.^9^ The same authors showed that Bi doping induces a broad photoluminescence band ranging between 440 nm (2.82 eV) and 550 nm (2.25 eV) associated with a progressive decrease in the host photoluminescence at 410 nm. As Mn is added to the material, a sharp emission at ∼600 nm appears in the red region. The simultaneous presence of Bi^3+^ and Mn^2+^ was shown to exhibit properties similar to the sum of those detected for the singly doped CsPbCl_3_ perovskite.^9,17^

Given the relevance of Mn/Bi co-doped CsPbCl_3_ perovskite for optoelectronics applications, we report here DFT calculations of individual Mn^2+^- and Bi^3+^-doped perovskites and those of co-doped systems to provide a quantitative understanding of the electronic and structural properties of these materials with inference to the possible carrier trapping at the dopant sites. We employ a state-of-the-art computational strategy combining hybrid DFT and spin–orbit coupling (SOC) that turns out to be crucial both for obtaining a proper energy level alignment and for obtaining reliable structural geometries.^48,49^ Our results show that Mn^2+^ can trap a hole and be consequently oxidized into its +3 state, whose state appears deep in the band gap. Notably, the transition energy for recombination of a conduction band electron and a Mn-trapped hole in Mn-doped CsPbCl_3_ almost coincides with that of the typical ^4^T_1_ → ^6^A_1_ transition related to the observed orange luminescence, possibly constituting an additional recombination pathway in the doped CsPbCl_3_ perovskite. The incorporation of Bi, similarly to what happens in MAPbI_3_,^22,23^ provides a trap state for electrons that may act as a recombination center with a valence band hole. We additionally investigated the role of the interaction between the two different dopants, simulating adjacent and non-adjacent doping lattice sites. We find that the electronic properties of the co-doped perovskite are not drastically affected by the separation between the Bi and Mn heterometals, suggesting negligible interaction between the two co-dopants.

Our computational setup delivers a 3.05 eV band gap for the pristine CsPbCl_3_ perovskite, in excellent agreement with the experimental value of 3.10 eV.^9,17^ The projected density of states (PDOS) reported in Figure 1 indicates that the description of the CsPbCl_3_ perovskite electronic structure is, as expected, similar to that of MAPbCl_3_.^50^ The valence band is characterized by a major contribution (∼66%) of Cl 3p orbitals (Figure 1a–b), with a significant involvement (∼32%) of Pb 6s orbitals (Figure 1a–c), while the conduction band is almost entirely contributed by Pb 6p orbitals.

**Figure 1:**
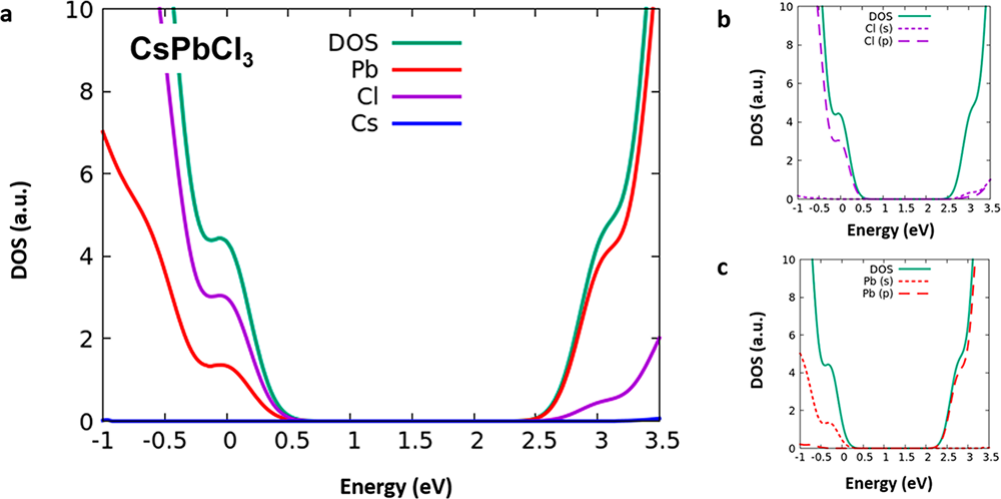
(a) Projected density of states of the pristine CsPbCl_3_ computed at the HSE06-SOC level of theory with partial Pb contributions (red), Cl contributions (purple), and Cs contributions (blue). (b) Contributions of Cl (s) and Cl (p) orbitals to the density of states of CsPbCl_3_. (c) Contributions of Pb (s) and Pb (p) orbitals to the density of states of CsPbCl_3_.

Compared to the prototypical MAPbI_3_ perovskite and related lead iodide perovskites, we notice an increased contribution of Pb 6s orbitals to the valence band in CsPbCl_3_. This is likely due to the stronger electron donation to Pb from chlorine compared to iodine, which increases the energy of the antibonding Pb 6s/Cl 3p combinations leading, together with band gap opening, to the emergence of occupied Pb 6s states.

Both Mn- and Bi-doped CsPbCl_3_ perovskites, investigated in the more stable 2+ and 3+ states, respectively, present a small band gap increase (∼0.1 eV) due to the structural distortion introduced by the heteroatoms (Table S1). While this effect could vanish in the limit of infinite dilution, i.e., at significantly lower defect densities, doping clearly introduces hole/electron trap states in the perovskite band gap that play a key role in the perovskite electronic properties. The Bi^3+^-doped CsPbCl_3_ perovskite presents an unoccupied state located 2.73 eV above the valence band (VB), and thus quite close to the conduction band (CB) of the pristine perovskite (3.04 eV), which is characterized by an antibonding combination of Bi 6p orbitals and Cl 3p states, as it is visible in the PDOS and in the isodensity plot of the corresponding single-particle orbital reported in panels b and c of Figure 2. When an electron is added to this system, it becomes trapped at the Bi^3+^ site, formally leading to a reduced Bi^2+^ center, with the corresponding occupied state now lying 2.17 eV above the VB (Figure 2e−f). Structural data suggest that the lattice does not undergo a significant change upon trapping, with Bi–Cl distances slightly increasing from ∼2.7 to ∼2.8 Å (Figure 2a,d). Notably, by neglecting the effect of SOC in the structural relaxation of the defect-trapped electron, we predicted a significant structural distortion involving the axial Bi–Cl distances, which increase from 2.72 to 3.13 Å (see Figure S1). This structural difference is due to different energetics of the singly occupied orbital where the added electron is located against the CB energy, which would be otherwise unoccupied at the PBE-SOC level of theory. Considering the anticipated impact of both SOC and hybrid functional in precisely tuning the perovskite CB and dopant energy levels, we further investigated the stability of the Bi-doped perovskite with an added electron by carrying out SOC-HSE06 energy evaluations along a linear path connecting the distorted (SR-) and undistorted (SOC-PBE) optimized geometries. This analysis shows a shallow potential energy profile with the SOC-HSE06 minimum located in an intermediate geometry between the SR-PBE and SOC-PBE, lying 0.06 eV below the former. Despite the flat energy surface, the different geometries have a significant impact on the singly occupied orbital representative of the Bi-trapped electron, whose energy decreases by as much as ∼0.7 eV when going from the SOC-PBE to the estimated SOC-HSE06 minimum. This result should be considered as a warning for PBE structural optimizations not predicting the correct geometries for carrier trapping/detrapping in metal halide perovskites. In fact, structural optimizations of neutral iodine vacancies in MAPbI_3_ incorrectly predict the formation of a Pb–Pb dimer, which is instead not favored by SOC-HSE06.^48^ This is not the case for Bi^3+^ where the corresponding orbital is unoccupied.

**Figure 2:**
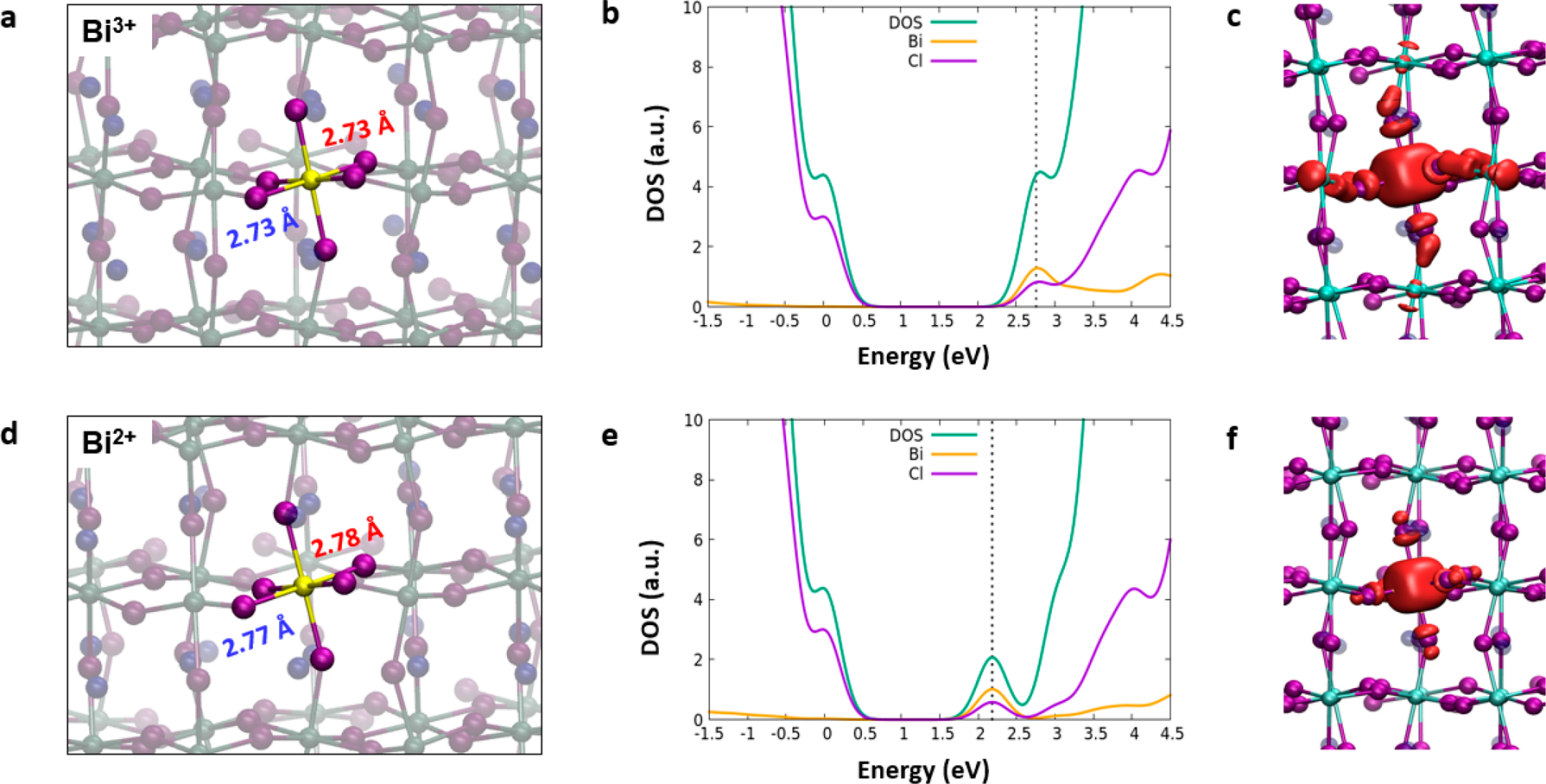
Main geometrical parameters calculated for (a) Bi^3+^ and (d) Bi^2+^ individually doped structures where equatorial distances are reported in blue and axial ones are reported in red. Projected densities of states (PDOS) computed for the singly doped (b) Bi^3+^ and (e) Bi^2+^ perovskites with dashed lines highlighting the states associated with the Bi. The diagrams are aligned with the CsPbCl_3_ pristine system using the 5d *j* = 1.5 orbital peak, and the energy reference (zero) is the VB of the pristine perovskite. Isodensity plots of the Kohn–Sham states located under the conduction band for the (c) oxidized and (f) reduced forms. Bi is colored yellow.

The electronic structure of the Mn^2+^-doped CsPbCl_3_ perovskite is not significantly different from that of the pristine material, with the exception of the aforementioned slight increase in the band gap. Occupied states related to Mn^2+^ are found deep in the VB. By order of decreasing energy, the first Mn contribution to the DOS starts ∼0.65 eV below the VB edge, being represented by partly hybridized Mn–Cl states (Figure 3b−c). These hybridized states extend for ∼2.5 eV, while a main peak related to the Mn 3d shell is found at a still lower energy. This is consistent with previous results for Mn-doped MAPbI_3_ employing the same level of theory^42^ but is at variance with the results of ref (39) showing in-gap Mn states, which were obtained by non-hybrid/non-SOC DFT. Similar results are indeed obtained by us by PBE (see Figure S2), clearly pointing to a significant interplay of exact exchange and SOC in determining the electronic properties of Mn-doped perovskites, consistent with ref (40), which found Mn unoccupied states buried in the CB when SOC was included. Also interesting is the fact that the extent of Mn hybridization with perovskite states seems to depend on the nature of the halide (see Figure S2). In the oxidized Mn^3+^ form, we notice the emergence of an unoccupied orbital, placed 0.87 eV above the VB (Table S1 and Figure 3e), which originates from an antibonding combination between a Mn state and chlorine p orbitals, as is shown by the isodensity plot reported in Figure 3c–f. A Jahn–Teller distortion in the equatorial plane occurs for Mn^3+^, which is signaled by the considerable decrease in the Mn–Cl bond length compared to the Mn^2+^ case, with a bond shortening from ∼2.6 to ∼2.4 Å (Figure 3a–d).

**Figure 3:**
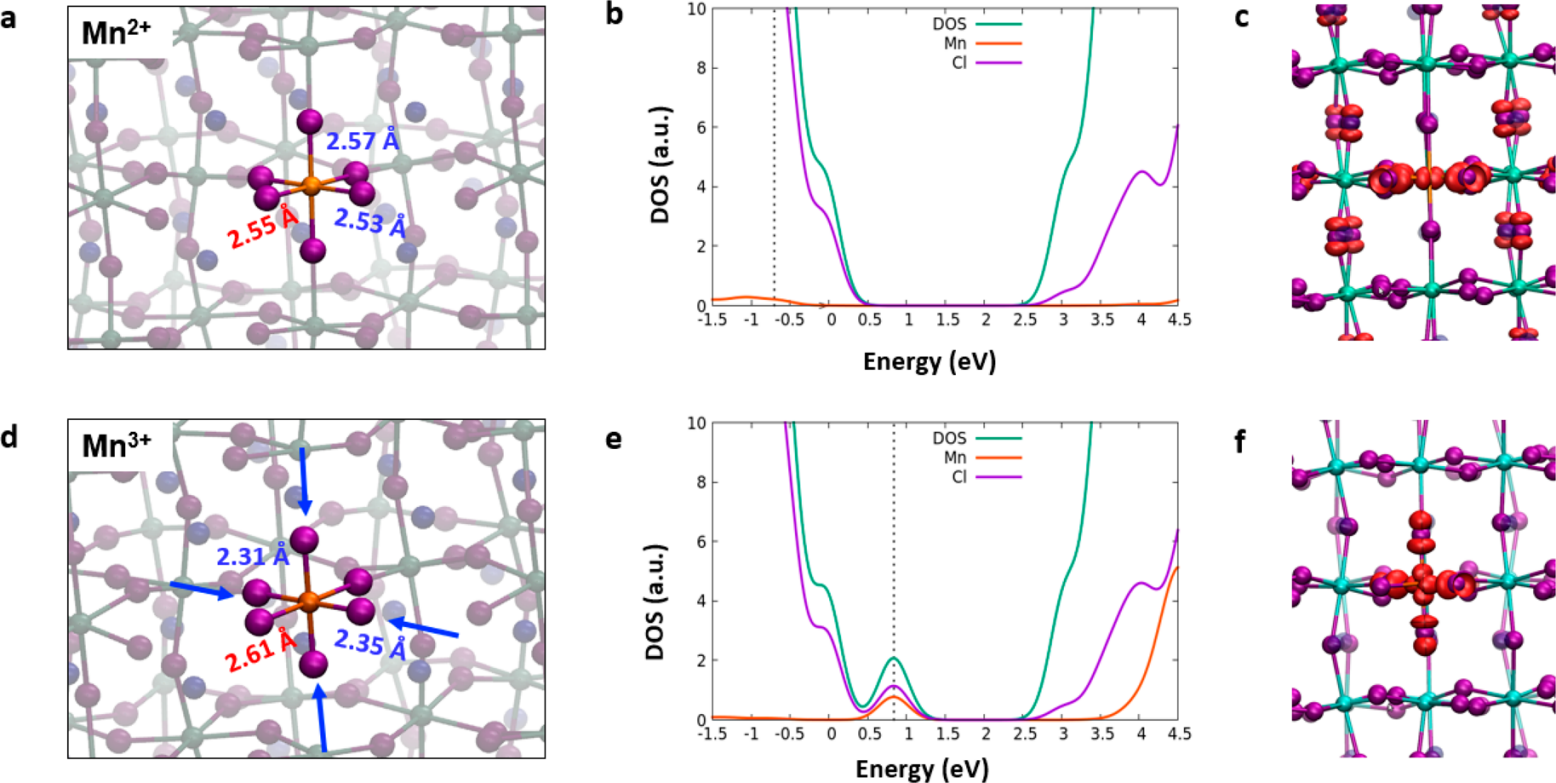
Main geometrical parameters calculated for (a) Mn^2+^ and (d) Mn^3+^ individually doped structures where equatorial distances are reported in blue and axial ones are reported in red. Projected density of states (PDOS) computed for the singly doped (b) Mn^2+^ and (e) Mn^3+^ perovskites with dashed lines highlighting the states associated with the Mn. The diagrams are aligned to the CsPbCl_3_ pristine system using the 5d *j* = 1.5 orbital peak, and the energy reference (zero) is the VB of the pristine. Isodensity plots of the Kohn–Sham states located in the band gap for the (c) reduced and (f) oxidized forms. Mn is colored orange.

We can now combine the electronic structure data described above to identify the possible trapping/detrapping pathways for non-interacting Bi^3+^- and Mn^2+^-doped CsPbCl_3_ (Figure 4). The presence of a Bi state below the CB (2.73 eV) traps the photoexcited electrons forming a Bi^2+^ species (Figure 4a). Subsequently, the electron may recombine with a hole in the VB and regenerate the Bi^3+^ species. This process is associated with an emission falling at 1.49–2.17 eV (SOC-HSE06 or SOC-PBE minimum), thus underestimating the experimental value of 2.6–2.8 eV.^9^ Closer agreement with the experiment is obtained if one considers a transition from the unoccupied state in the gap (∼2.7 eV), although emission should be accompanied by occupation of the state and its associated structural relaxation, so unless nonthermalized emission takes place, the former values should be representative of the process.

**Figure 4:**
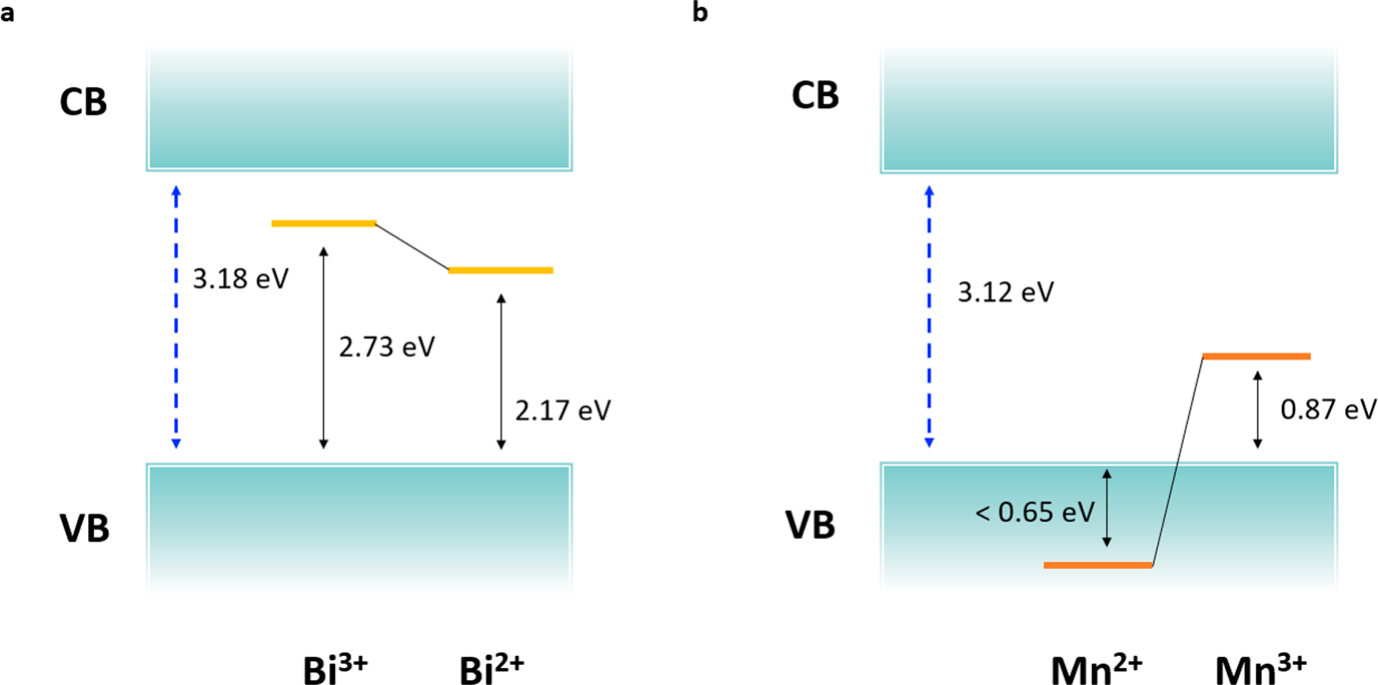
(a) Electron trapping pathway in Bi^3+^-doped CsPbCl_3_ perovskite and (b) hole trapping pathway in Mn^2+^-doped CsPbCl_3_ perovskite.

For the Mn^2+^-doped perovskite, our analysis suggests the process schematized in Figure 4b. After electronic excitation, hole trapping at Mn^2+^ leads to geometrical distortion due to the Jahn–Teller effect on the Mn^3+^ site that causes the emergence of a hole state located 0.87 eV above the VB. This state may provide a possible de-excitation pathway by recombining with a CB electron with an associated transition energy of 2.21 eV, which is close to the experimental value of ∼2.1 eV.^17,48^ We notice, however, that most of the literature on Mn^2+^-doped semiconductors agrees on such luminescence as originating by the crystal field ^4^T_1_ → ^6^A_1_ transition of the Mn^2+^ ion, populated by energy transfer from the host to the Mn^2+^ ion.^15^ In this case, the energy of the ^4^T_1_ → ^6^A_1_ transition related to the luminescence, experimentally observed at 2.1 eV, can be overlapping with the transition corresponding to hole trapping at Mn^2+^, thus constituting a possible additional recombination channel. We notice that the contribution of the transient Mn^3+^ intermediate state precursor to the ^4^T_1_ → ^6^A_1_ luminescence has been very recently documented in Mn^2+^-doped CdZnSe quantum dots,^37^ suggesting a similar intermediate process taking place in CsPbCl_3_, as well.

To understand if the presence of the two dopants in the same CsPbCl_3_ perovskite host would influence the electronic properties of the material, we investigated Mn/Bi co-doped crystals where the two metals are placed in a non-interacting position lying on two different layers of the lattice at a distance of ∼12 Å and in two adjacent octahedra (see Figure 5a−b). We then computed the Mn^3+^/Bi^3+^ and Mn^2+^/Bi^3+^ co-doped perovskites for both the interacting and non-interacting cases. We found only a slight variation of the energy levels [∼0.1 eV at most (see Figure 6)], suggesting that singly doped systems are reliable models of the Mn/Bi co-doped systems. Furthermore, the concomitant presence of the two heterometals in an interacting position at a relative distance of ∼6 Å (Figure 5b) does not significantly alter the electronic properties (compare panels c and d of Figure 6 to panels a and b of Figure 6) or the relative stability of the two systems, with the interacting Mn^2+^/Bi^3+^ configuration lying within 0.02 eV with respect to the non-interacting Mn^2+^/Bi^3+^ one. These findings are in agreement with the experimental data where the photoluminescence observed in co-doped nanocrystals is essentially unmodified with respect to that observed in individually doped systems.^9,17^

**Figure 5:**
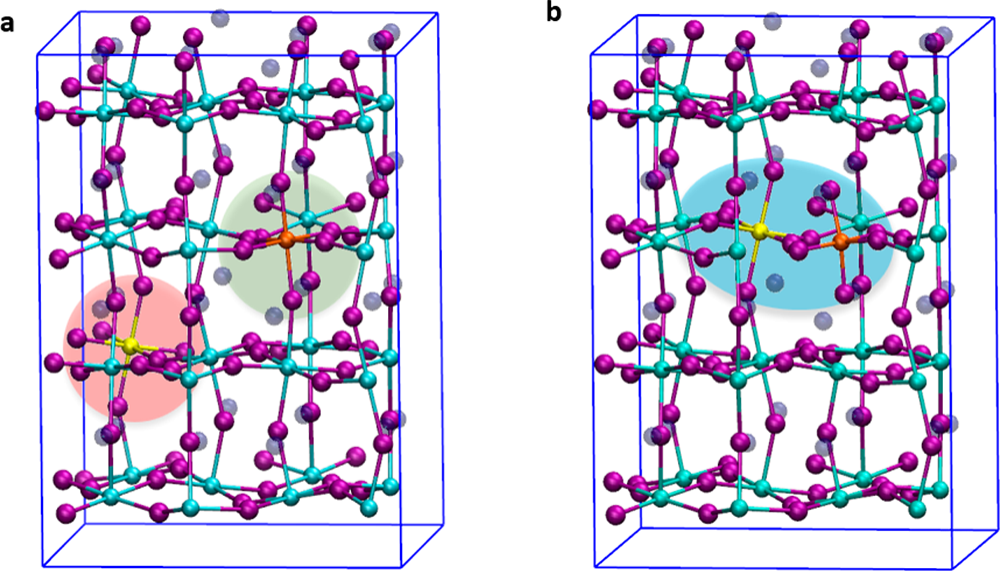
Supercells employed for the modeling of (a) non-interacting and (b) interacting Mn/Bi CsPbCl_3_ co-doped perovskites. Mn is colored orange, and Bi yellow.

**Figure 6:**
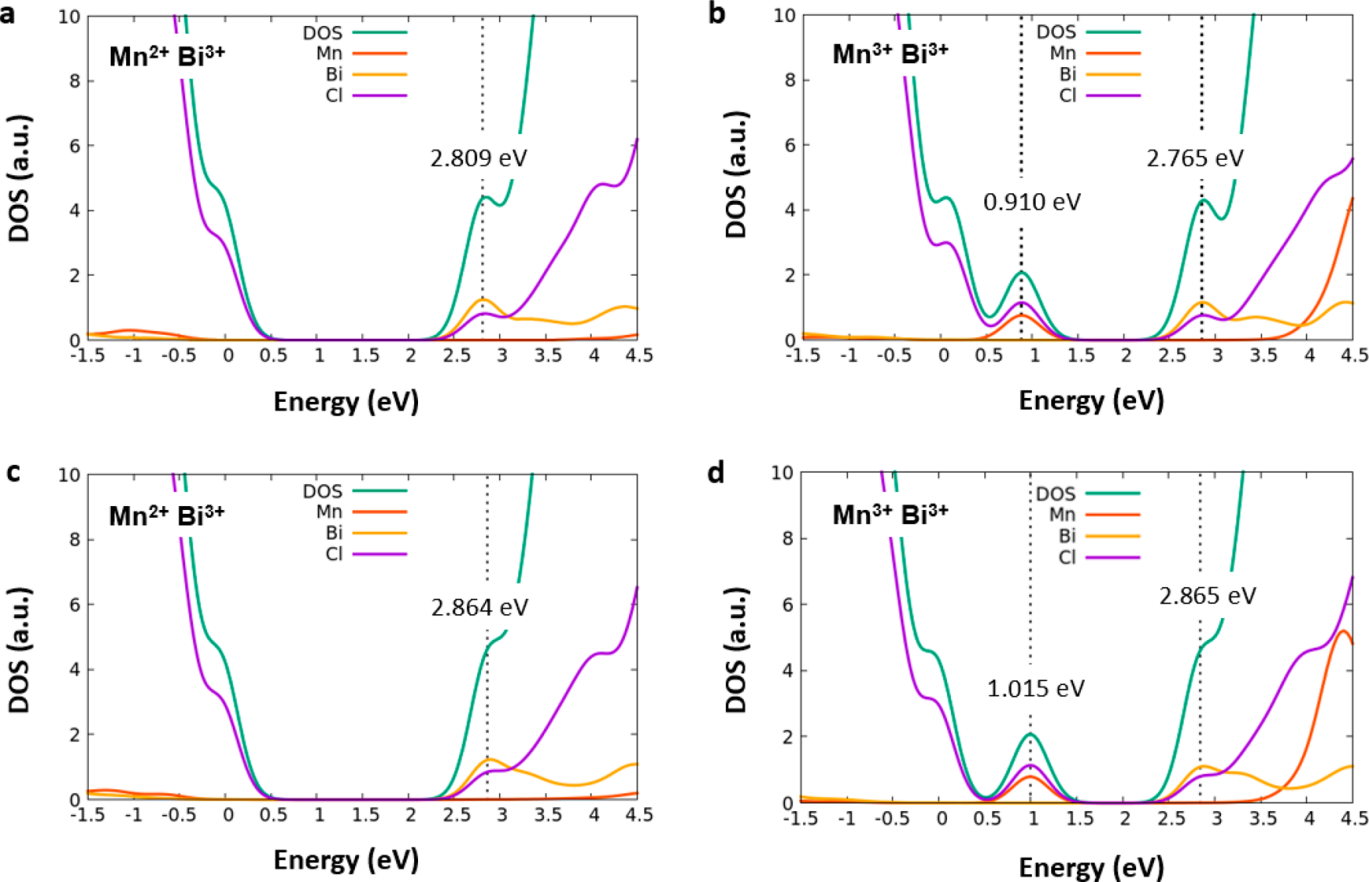
Projected densities of states (PDOS) computed for the non-interacting co-doped (a) Mn^2+^/Bi^3+^ and (b) Mn^3+^/Bi^3+^ perovskites and for interacting (c) Mn^2+^/Bi^3+^ and (d) Mn^3+^/Bi^3+^ ones. The PDOS diagrams are aligned with the CsPbCl_3_ pristine system using the 5d *j* = 1.5 orbital peak, and the energy of the VB of the pristine is set to zero.

The simultaneous presence of Bi^3+^ and Mn^2+^ thus shows properties similar to the sum of those detected for the singly doped CsPbCl_3_ perovskite without significant interaction effects of the two dopant sites. This can be visualized in the global dopant energy levels of Figure 7.

**Figure 7:**
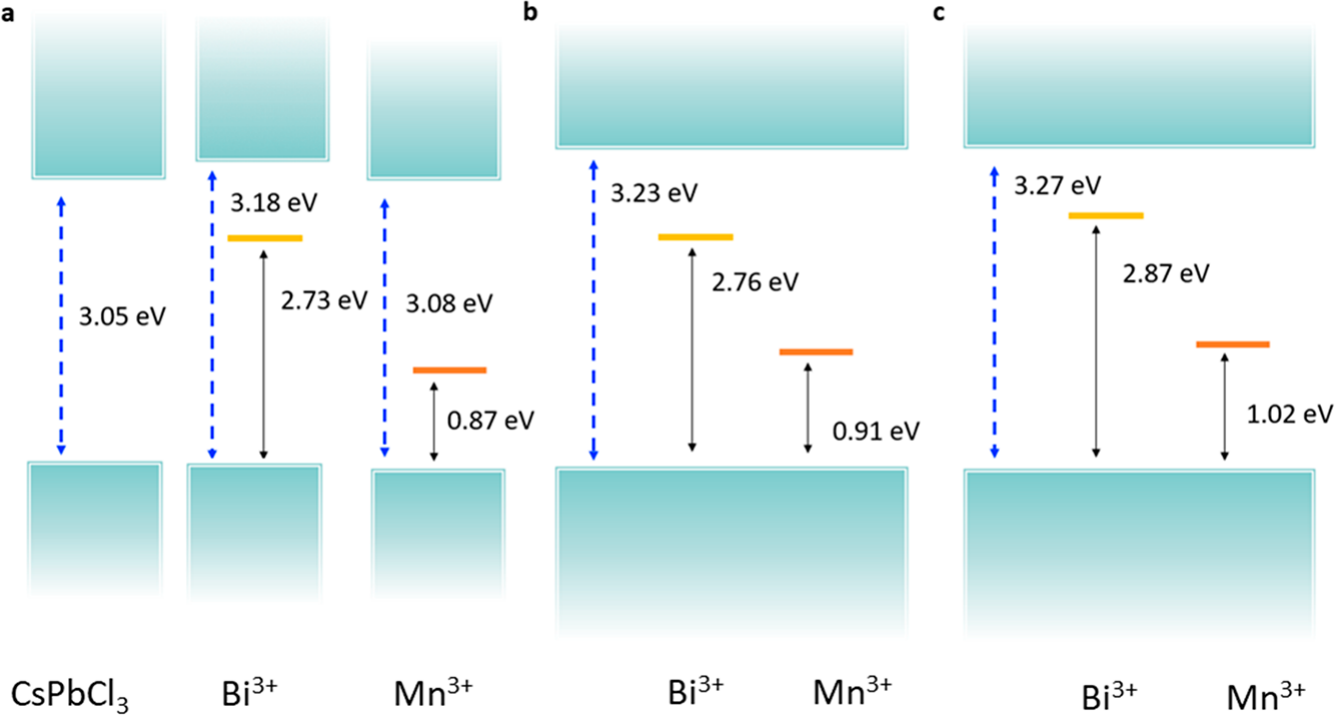
Graphical representation of band edge and dopant trap states for CsPbCl_3_ individually doped by (a) Bi^3+^ and Mn^3+^ and Mn^3+^/Bi^3+^ co-doped in (b) non-interacting and (c) interacting configurations.

In summary, we calculated the structural and electronic properties of singly doped and co-doped Bi/MnCsPbCl_3_ perovskites by state-of-the-art first-principles calculations. We find a significant combined effect of spin–orbit coupling and hybrid functional on structural features of electrons trapped at the Bi dopant site, with scalar relativistic geometry optimization leading to large structural deformation. Electron trapping at the Bi dopant site introduces a defect state within the material band gap, as well as hole trapping at Mn. A significant Jahn–Teller distortion occurs upon hole trapping at the Mn^2+^ defect, while the extent of structural relaxation upon electron trapping at Bi depends on the considered level of theory, SOC-HSE06 results delivering a partial distortion. The energy of the ^4^T_1_ → ^6^A_1_ transition related to the typical luminescence related to Mn doping in conventional semiconductors is overlapping with the transition corresponding to hole trapping at Mn^2+^, thus constituting an additional recombination channel in the CsPbCl_3_ perovskite. A possible role of Mn^3+^ in mediating excitation transfer from the perovskite host to the excited Mn^2+^ dopant is proposed, which may constitute an additional recombination channel for photogenerated charge carriers.

## Computational Details

The equilibrium structures of individually doped, co-doped, and pristine CsPbCl_3_ perovskites were modeled in a 2 × 2 × 2 supercell with a total of 160 atoms, employing the tetragonal structure of the material, using the Perdew–Burke–Ernzenhof (PBE) exchange-correlation functional^51^ including scalar relativistic (SR-PBE) and spin–orbit corrections (SOC-PBE) and relaxing ion positions until forces on atoms were less than 0.001 Ry Å^–1^. All PBE calculations were performed with ultrasoft pseudopotentials and the plane wave basis set implemented in the Quantum Espresso Program Package.^52^ Cutoffs on the plane waves and the charge density of 25 and 200 Ry, respectively, were used, sampling the Brillouin zone at the k-point Γ. The dopants, Mn and Bi, were disposed in a substitutional position, in place of the metal lead, assuming a doping concentration of 3.12%, and the supercell was relaxed with the same procedure explained previously with fixed cell parameters. In the case of co-doped perovskite, structures with different relative positions of the two substitutional cations were considered, simulating interacting and non-interacting dopants. To investigate the optical properties of the systems, all PBE-SOC geometries involving pristine, doped, and co-doped perovskites were refined employing the Heyd-Scuseria-Ernzerhof 2006 (HSE06) hybrid functional,^53^ including spin–orbit coupling corrections. Norm-conserving (NC) pseudopotentials were used with a cutoff on the wave function of 40 Ry and a cutoff on the Fock grid of 80 Ry, sampling at the Γ point of the Brillouin zone. An increased fraction of exact exchange has been included in the HSE06 functional α = 0.43. It has been shown that this computational setup provides accurate band gaps for MAPbI_3_ perovskites compared to the experiment and to accurate GW calculations,^49^ and it is normally employed for the quantitative prediction of thermodynamic ionization levels of defects in these materials.^48,54^

## Supporting Information Available

Aligned energy levels and SR-PBE relaxed structures and DOS (PDF)
