# Supplementary material for: Electronic Properties and Carrier Trapping in Bi and Mn Co-doped CsPbCl3 Perovskite
Source: J Phys Chem Lett. 2020 Jun 17;11(14):5482–9. doi: 10.1021/acs.jpclett.0c01567 (PMC8008448; doi:10.1021/acs.jpclett.0c01567)
Supplement: Supplementary file 1 — jz0c01567_si_001.pdf [file jz0c01567_si_001.pdf]

Supporting Information (SI)

Electronic Properties and Carrier Trapping in Bi and Mn co-doped $CsPbCl_3$ Perovskite

Damiano Ricciarelli,[a,b] Edoardo Mosconi,[b] * Boualem Merabet,[c,d] Olivia Bizzarri,[b] Filippo De Angelis[a,b,e] *

[a] Department of Chemistry, Biology and Biotechnology, University of Perugia, Via Elce di Sotto 8, 06123 Perugia, Italy.
[b] Istituto CNR di Scienze e Tecnologie Chimiche "Giulio Natta" (CNR-SCITEC), Via Elce di Sotto 8, 06123 Perugia, Italy.
[c] Faculty of Sciences and Technology, University of Mustapha Stambouli, Mascara 29000, Algeria.
[d] Laboratoire de Physique Computationnelle des Materiaux , Faculté de Sciences Exates, Département de Physique, Université Djillali Liabès, Sidi Bel Abbès 22000, Algeria.
[e] CompuNet, Istituto Italiano di Tecnologia, Via Morego 30, 16163 Genova, Italy.

Table S1. Band edges and dopant deep Kohn-Sham states of MnBi individual and co-doped $CsPbCl_3$ perovskites. The energy values are aligned to the $CsPbCl_3$ pristine system using the 5d j=1.5 orbital peak and the energy reference (zero) is the VB of the pristine. We also specify where the dopant deep states are mainly localized and whether they are occupied or unoccupied.

	**Valence band (eV)**	**Dopant deep states (eV)**	**Conduction band (eV)**	**Band gap (eV)**
**Pristine**	0.000	-	3.05	3.05
**Mn^{2+}**	-0.05	-	3.07	3.13
**Mn^{3+}**	-0.04	0.84 (Mn unocc.)	3.05	3.08
**Bi^{3+}**	0.03	2.76 (Bi unocc.)	3.21	3.18
**Bi^{2+} (SOC-PBE optimization)**	0.02	2.19 (Bi occ.)	3.09	3.07
**Bi^{2+} (SR-PBE optimization)**	-0.06	1.11 (Bi occ.)	3.03	3.09
**$Mn^{2+}Bi^{3+}$ (non-interacting)**	-0.05	2.81 (Bi unocc.)	3.28	3.32
**$Mn^{3+}Bi^{3+}$ (non-interacting)**	-0.05	0.91 (Mn unocc.)	3.24	3.28

		2.77 (Bi unocc.)		
**$Mn^{2+}Bi^{3+}$ (interacting)**	-0.05	2.86 (Bi unocc.)	3.27	3.31
**$Mn^{3+}Bi^{3+}$ (interacting)**	-0.03	1.00 (Mn unocc.)<br>2.84 (Bi unocc.)	3.25	3.28

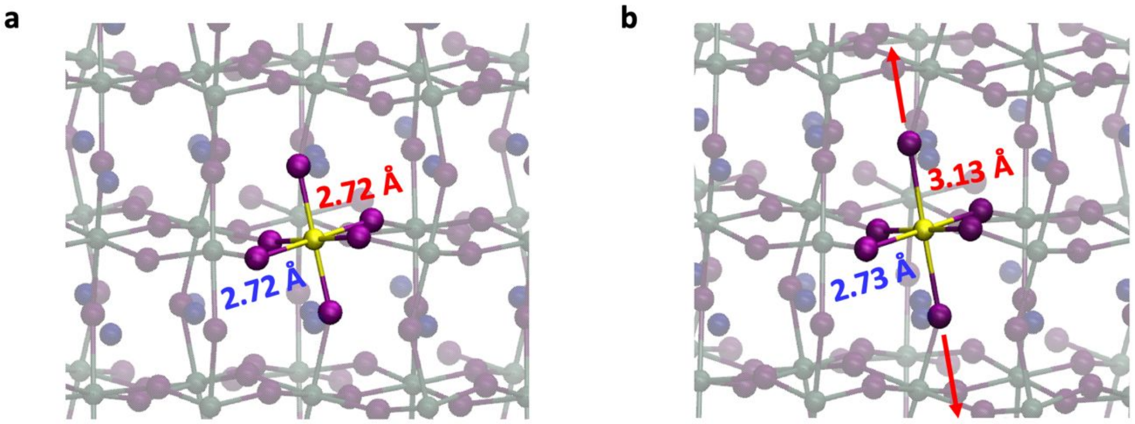

Figure S1. Geometrical structures obtained for Bi^{3+} (a) and Bi^{2+} (b) individual doped perovskites at the PBE level of theory. Equatorial distances are reported in blue while axial ones in red.

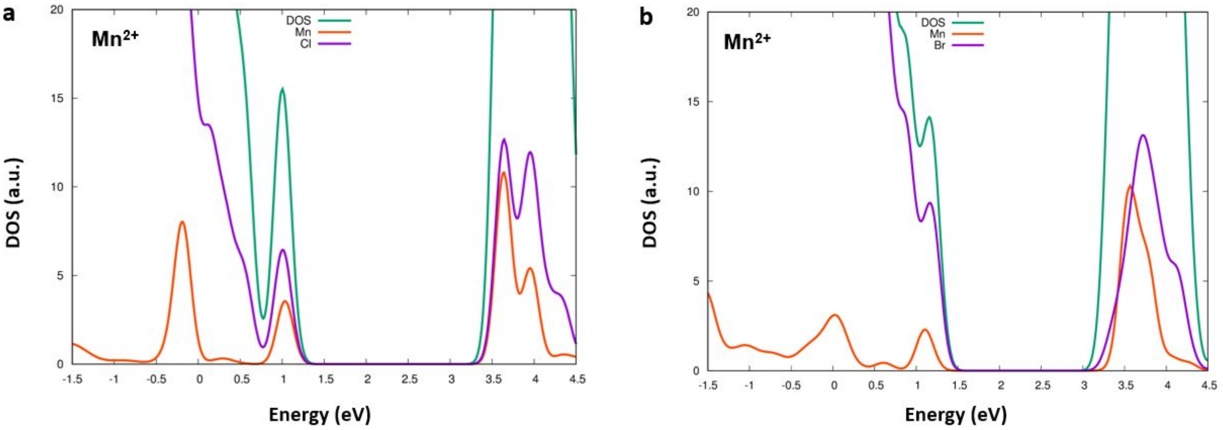

Figure S2. Projected density of states (PDOS) computed for the individual doped Mn^{2+} $CsPbCl_3$ (a) and $MAPbBr_3$ (b) at PBE level of theory. The energy was aligned to the pristine $CsPbCl_3$ computed at the HSE06(0.43)-SOC level of theory and the reference for energy is the valence band of the former.
